# Effects of amiodarone on short QT syndrome variant 3 in human ventricles: a simulation study

**DOI:** 10.1186/s12938-017-0369-0

**Published:** 2017-06-07

**Authors:** Cunjin Luo, Kuanquan Wang, Henggui Zhang

**Affiliations:** 1 School of Computer Science and Technology, Harbin Institute of Technology (HIT), Harbin, 150001 China; 2 School of Physics and Astronomy, The University of Manchester, Manchester, M13 9PL UK; 3 Space Institute of Southern China, Shenzhen, 518117 China

**Keywords:** Arrhythmia, Short QT syndrome, Computer simulation, *KCNJ2*, D172N, Amiodarone

## Abstract

**Background:**

Short QT syndrome (SQTS) is a newly identified clinical disorder associated with atrial and/or ventricular arrhythmias and increased risk of sudden cardiac death (SCD). The SQTS variant 3 is linked to D172N mutation to the *KCNJ2* gene that causes a gain-of-function to the inward rectifier potassium channel current (*I*_K1_), which shortens the ventricular action potential duration (APD) and effective refractory period (ERP). Pro-arrhythmogenic effects of SQTS have been characterized, but less is known about the possible pharmacological treatment of SQTS. Therefore, in this study, we used computational modeling to assess the effects of amiodarone, class III anti-arrhythmic agent, on human ventricular electrophysiology in SQT3.

**Methods:**

The ten Tusscher et al. model for the human ventricular action potentials (APs) was modified to incorporate *I*_K1_ formulations based on experimental data of Kir2.1 channels (including WT, WT-D172N and D172N conditions). The modified cell model was then implemented to construct one-dimensional (1D) and 2D tissue models. The blocking effects of amiodarone on ionic currents were modeled using IC_50_ and Hill coefficient values from literatures. Effects of amiodarone on APD, ERP and pseudo-ECG traces were computed. Effects of the drug on the temporal and spatial vulnerability of ventricular tissue to genesis and maintenance of re-entry were measured, as well as on the dynamic behavior of re-entry.

**Results:**

Amiodarone prolonged the ventricular cell APD and decreased the maximal voltage heterogeneity (*δV*) among three difference cells types across transmural ventricular wall, leading to a decreased transmural heterogeneity of APD along a 1D model of ventricular transmural strand. Amiodarone increased cellular ERP, prolonged QT interval and decreased the T-wave amplitude. It reduced tissue’s temporal susceptibility to the initiation of re-entry and increased the minimum substrate size necessary to sustain re-entry in the 2D tissue.

**Conclusions:**

At the therapeutic-relevant concentration of amiodarone, the APD and ERP at the single cell level were increased significantly. The QT interval in pseudo-ECG was prolonged and the re-entry in tissue was prevented. This study provides further evidence that amiodarone may be a potential pharmacological agent for preventing arrhythmogenesis for SQT3 patients.

**Electronic supplementary material:**

The online version of this article (doi:10.1186/s12938-017-0369-0) contains supplementary material, which is available to authorized users.

## Background

Short QT syndrome (SQTS) is a potentially lethal cardiac ion channelopathy associated with a variety of signs and symptoms, from dizziness and fainting (syncope) to cardiac arrest and sudden death [1–6]. These signs and symptoms can occur anytime from early infancy to old age. Among which, short QT interval in SQTS patients is commonly less than 360 ms, and T-wave morphology is tall and symmetrical. SQTS with various mutations is genetically heterogeneous, involving the *KCNH2* [4], *KCNQ1* [3], *KCNJ2* [2], *CACNA1C* [7], *CACNB2b* [7] and *CACNA2D1* [8] genes. These identified gene mutations in membrane ion channels alter the electrical activity of the heart, leading to arrhythmic characteristic of SQTS. Among the mutations, either a gain-in-function of the potassium channel or a loss-in-function of the calcium channel across the membrane of cardiac muscle cells has been observed [2–4, 7, 8].

The SQTS is associated with accelerated ventricular repolarization and with an increased risk of atrial and/or ventricular arrhythmias and of sudden cardiac death (SCD) [6, 9]. A gain-of-function *KCNJ2* mutation has been identified in SQTS variant 3 (SQT3), with a 5-year-old child and her father exhibiting a corrected QT interval (QTc) of 315 and 320 ms, respectively, and a narrow and peaked T-wave morphology on the clinical electrocardiogram (ECG) [2]. Based on a systematic genetic analysis, a pair of complementary bases substituted from aspartic acid to asparagine at position 172 in the Kir2.1 channel was discovered. The results from in vitro voltage clamp recordings have demonstrated an increased *I*_K1_ in the Kir2.1 channels during terminal repolarization of the action potentials (APs) [2]. Our previous computational studies [10] have shown that SQT3 abbreviated the AP duration (APD) and effective refractory period (ERP) and steepened APD restitution (APD-R) and ERP restitution (ERP-R) curves, which consequently shortened the QT interval and raised the T-wave amplitude. Whereas pro-arrhythmogenic effects of SQT3 in the human ventricles have been investigated [2, 10], less is known about the pharmacological agents that prevent incidence of arrhythmogenesis in SQT3 patients.

Currently, the only proven therapy for SQTS patients is the application of an electric shock, delivered via an implantable cardioverter defibrillator (ICD) [9, 11–13]. Despite the success of this technique, there is an increased risk of an inappropriate shock discharged by the ICD [9, 11, 12, 14]. As the QT interval is not restored within the normal range over time, ICD is unsuitable for SQTS patients [12]. Although SQTS patients may benefit from subcutaneous ICD, additional cautions are needed to affirm the safety of such device [15]. To effectively treat SQTS patients, pharmacological therapy may be the primary modality to restore the normal QT interval and protect against arrhythmia [9, 11, 16, 17]. However, data regarding pharmacological treatment of SQTS are very limited. A clinical study by Lu et al. [18] showed that amiodarone prevented the incidence of arrhythmia and prolonged the QT interval in an SQTS patient, but electrophysiologic testing data were not available in that patient. Long-term therapy of amiodarone may be associated with unwanted effects. Important side effects were seen in the thyroid gland, liver, lung and skin. Some of these side effects are dose-dependent, and others may be related to the chemical structure and metabolism of amiodarone [19]. At present, there is no experimental model of SQT3. Moreover, drugs exert effects spanning from the ion channel to the whole organ level, making investigation using experimental techniques tough and rather expensive [20]. In recent years, computational models of the heart are beginning to play a critical role in the investigations of arrhythmias and anti-arrhythmic therapy. Therefore, this study was undertaken in order to assess the effects of amiodarone, a class III anti-arrhythmic agent, on human ventricular cell and tissue model in SQT3 conditions, and consequent effects of amiodarone on QT interval prolongation and prevention of re-entrant arrhythmia in this form of SQTS.

## Methods

**Fig. 1:**
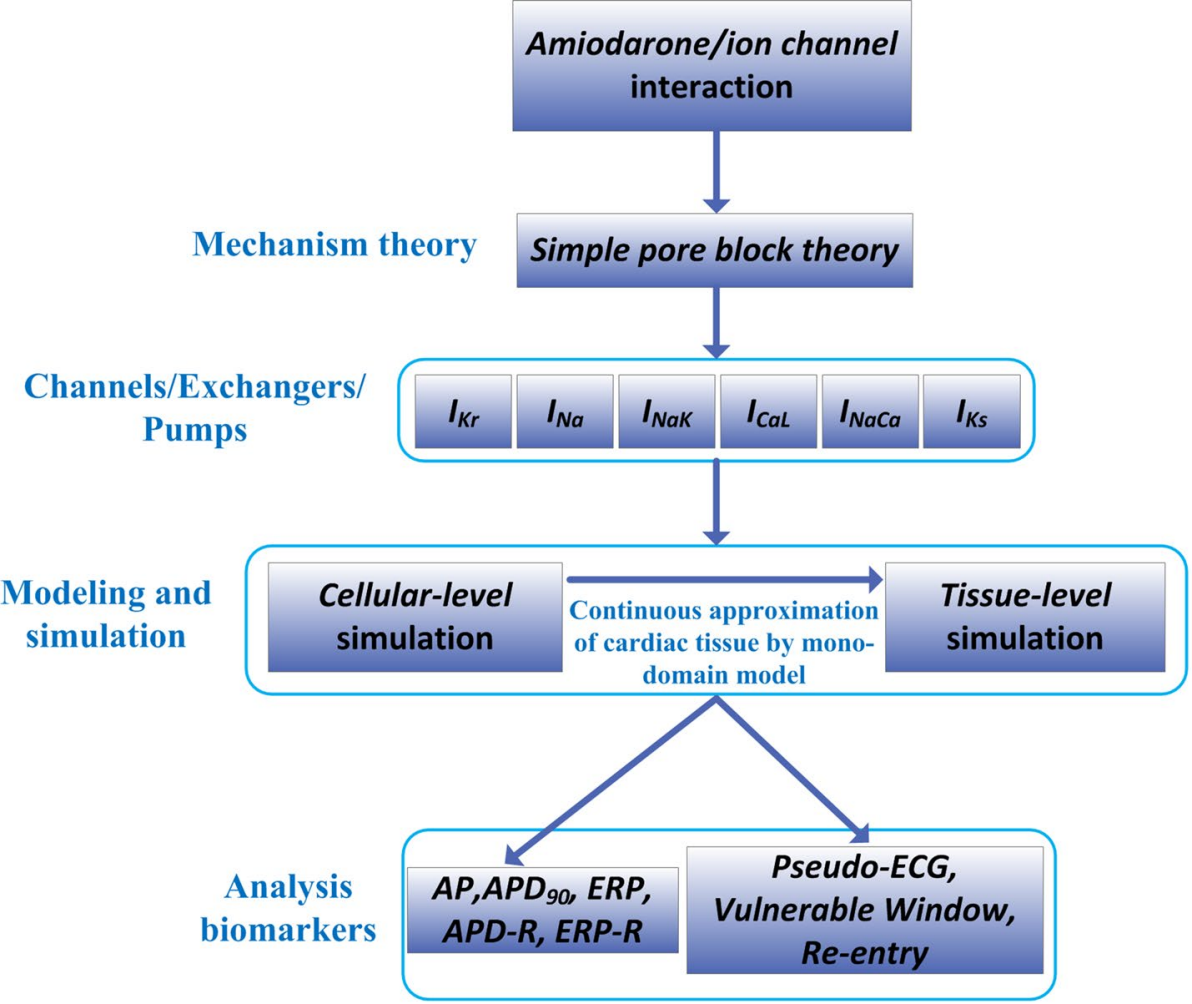
Computer-based assessment flowchart for testing amiodarone actions at hierarchical levels including ion channel, cellular and tissue. The actions of amiodarone on ventricular excitation associated with SQT3 at cell and tissue levels can be characterized by analyzing their effects on a family of biomarkers

We proposed an assessment flow for evaluating the actions of amiodarone on ventricular excitation associated with SQT3 at the cell and tissue levels using mathematical models of the heart, as illustrated in Fig. 1.

### *I*_K1_ kinetics and drug/ion current channel interaction

The ten Tusscher et al. [21] model for human ventricular endocardial (ENDO), mid-myocardial (MIDDLE) and epicardial (EPI) APs was used for this study. The parameters of *I*_K1_ model were modified to incorporate the experimentally observed kinetic properties of the wild-type (WT) and mutant homozygous (D172N) and heterozygous (WT-D172N) *I*_Kir2.1_. The *I*_K1_ model formulation is described as

1 $$I_{K1} = G_{K1} \sqrt {\frac{{K_{o} }}{5.4}} x_{K1\infty } (V - E_{K} )$$

where *V* is the transmembrane voltage, *E*_K_ is the potassium reversal potential, *G*_K1_ is the maximum conductance, and *x*_k1∞_ is a rectification factor, which is a characteristic of the open-up probability of the channel. The *I*_K1_ model used was that of ten Tusscher [21], which was based on the formulation of Priebe and Beuckelmann [22] model. Experimentally obtained current–voltage (*I*–*V*) data [23] were used to modify the *I*_K1_ formulations for WT, WT-D172N and D172N conditions. This was achieved by simulating the experimental voltage-clamp protocol [23], with which the experimental data [23] were fitted to model equations by using the Broyden-Fletcher-Goldfarb-Shanno optimization algorithm following our previous study [10]. Relative current proportions for WT, WT-D172N and D172N conditions were scaled by using relative proportions of peak *I*_Kir2.1_ [23]. The peak *I*_Kir2.1_ was 4.6-fold for the D172N condition and 2.2-fold for the WT-D172N condition than that for the WT condition [23]. In simulations, Eq. 1 was modified to incorporate the D172N-induced changes in the channel kinetic properties, including (i) the marked augmentation of an outward Kir2.1 current through the D172N channel, and (ii) the rightward voltage shift of the peak repolarization current. The modified *I*_K1_ formulations for WT, WT-D172N and D172N conditions [10] are shown in Additional file 1.

A simple pore block theory [24] was used in this study to model the drug/ion channel interactions. With this theory, the effect of a drug on blocking an ion channel can be simulated using a blocking factor *θ* that reduces the maximal conductance of an ionic current is modified in a dose-dependent manner such that:

2 $$\theta = \frac{1}{{1 + \left( {\frac{{IC_{50} }}{[D]}} \right)^{nH} }}$$

where [*D*] is the concentration of a drug, IC_50_ is the half maximal inhibitory concentration, and nH is the Hill coefficient. The respective reduction of ionic currents in the presence of amiodarone was determined by using IC_50_ and nH values as shown in Table 1. In this study, two concentrations of amiodarone in the effective therapeutic range were chosen. The range of clinical therapeutic concentrations is between 1 μM (low concentration) and 3 μM (high concentration) [25, 26]. The resulting ion channel conductivity reductions relative to their original values in the presence of amiodarone are provided in Table 2.

**Table 1:** Inhibition of cardiac ion currents in the presence of amiodarone

Drug	Amiodarone					
Current	*I*_Kr_	*I*_Na_	*I*_NaK_	*I*_CaL_	*I*_NaCa_	*I*_Ks_
IC_50_ (µM)	2.80	4.84	15.60	5.80	3.30	3.84
nH	0.91	0.76	1.00	1.00	1.00	0.63
Source	[27]	[28]	[29]	[30]	[31]	[32]

Data used in this study showing inhibition of ion currents in the presence of amiodarone. Half-maximal inhibitory concentration (IC_50_) and Hill coefficient (nH) values for amiodarone extracted from literatures

**Table 2:** Ion channel conductivities (% of original value) in the presence of amiodarone

Conductivity	Amiodarone					
	*G*_Kr_	*G*_Na_	*P*_NaK_	*G*_CaL_	*P*_NaCa_	*G*_Ks_
Low dose (1 µM)	71.28	76.33	93.98	85.29	76.74	69.42
High dose (3 µM)	48.41	58.78	83.87	65.91	52.38	53.73

Ion channel conductivities (% of original value) in the presence of amiodarone. Definition of amiodarone concentrations: low (1 μM) and high (3 μM)

### Single-cell model and AP simulations

In this study, the modified *I*_K1_ model was incorporated into the ten Tusscher et al. [21] model for the human ventricular cell AP. In the single cell AP model, the electrophysiological activity during different phases can be described by the following equation:

3 $$\frac{dV}{dt} = - \frac{{I_{ion} + I_{stim} }}{{C_{m} }}$$

where *V* is the transmembrane voltage, *t* is time, *C*_m_ is the cellular capacitance per unit surface area, *I*_stim_ is the external stimulus current, and *I*_ion_ is the sum of transmembrane ionic currents. The component of the late sodium current (*I*_NaL_) from the ORd model [33] was incorporated. The physical units are as follows: time *t* is in ms, potential *V* is in mV, current *I*_ion_ and externally applied stimulus current *I*_stim_ are in pA, current densities are in pA/pF, capacitance *C*_m_ is in μF/cm^2^, channel conductance *G* is in nS/pF, intra- and extracellular ionic concentrations are in millimolar. The code for the cell model was downloaded from http://www-binf.bio.uu.nl/khwjtuss/SourceCodes/. The cell model was paced with an S1–S2 protocol. An S1 was applied with a basic cycle length (BCL) of 800 ms and an amplitude of −52 pA/pF for 1 ms. An S2 with the same amplitude and duration as the S1 was applied after the AP evoked by S1. AP model was stimulated by the application of pulses of a 1.25 Hz frequency, which roughly corresponds to 75 bpm of normal human heart. Equation 3 was integrated by using the forward Euler method with a time step of 0.02 ms. The Hodgkin–Huxley (HH) equations for the gating variables of the various time-dependent currents including the modified *I*_K1_ in the ten Tusscher et al. model were integrated using the Rush and Larsen scheme [34].

### Tissue simulations

Initiation and conduction of APs in the multicellular tissue model were modeled with the following equation:

4 $$\frac{\partial V}{\partial t} = - \frac{{I_{ion} + I_{stim} }}{{C_{m} }} + \nabla \cdot (D\nabla V)$$

where *D* is the diffusion coefficient between ventricular cells describing the tissue’s electrical conductivity, and ∇ is the gradient operator.

For one-dimensional (1D) simulations, a single fiber model of transmural strand with a length of 15 mm was used (discretized into 100 nodes). The total length is close to the normal range of the human transmural ventricle wall thickness of ~4.0 to 14.0 mm [35]. The fiber employed a spatial resolution of 0.15 mm, with each node representing a 150-μM cylindrical cell. The fiber model was composed of three regions containing: ENDO, MIDDLE and EPI cells (with respective proportions of 25%: 35%: 40%). The lengths of each region were 3.75, 5.25 and 6 mm for ENDO, MIDDLE and EPI, respectively. These proportions and the total length are similar to those used in other studies [10, 36–38]. The diffusion coefficient, *D* was set at 0.0008 cm^2^/ms giving a conduction velocity (CV) of 52 cm/s through the fiber, which is relatively close to the experimental CV of ~50 cm/s [39, 40]. In the model, a fivefold decrease in gap junctional conductance between the EPI and MIDDLE region was implemented. This followed the study of Gima and Rudy [41], which was based on experimental work of Yan et al. [35] showing a sharp transition in the tissue resistance in a left ventricular wedge, implying reduced electrical coupling in this region [42].

The pseudo-ECG was computed as an integral of the spatial gradient of transmembrane potential of cells at all positions on the fiber from an electrode by using [41]:

5 $$\phi_{e} (x^{\prime }) = \frac{{\alpha^{2} }}{4}\int {( - \nabla V) \cdot \left[ {\frac{1}{r}} \right]dx}$$

where *∅*_*e*_ is the computed potential at the electrode, *α* is the radius of the strand, *r* is the Euclidean distance from a source point *x* to the electrode point *x*′, and *dx* is the spatial resolution. In this study, we placed the electrode at a position 2.0 cm from the ENDO end of the strand, which resulted in a negative QRS and a positive T wave in the pseudo-ECG waveforms. These waveforms were in concordance with the clinical data [2]. The QT interval was computed as the time interval determined by the definition of the onset of the QRS complex (*Q*_onset_ = 0.0 mV, *t* = 0 ms) and the point at which the end of the T-wave (*T*_end_) crossed the baseline. The time at which the ECG signal fell below a threshold (*V*_thresh_ = 0.01 mV) was defined as *T*_end_.

For two-dimensional (2D) simulations, an idealized geometry was implemented. The idealized geometry was a simple sheet of tissue measured 15 mm by 75 mm. It was modeled by expanding the 1D fiber (100 cells, 15 mm in the *x*-direction) into a sheet with a width of 75 mm in the *y*-direction. The spatial resolution in both *x* and *y* directions was the same as used in the 1D fiber. In the 2D sheet tissue, re-entry was initiated by an S1-S2 stimulation protocol. A planar wave was initiated at the ENDO end by an S1 stimulus (amplitude: −52 pA/pF, duration: 1 ms). During the vulnerable window, an S2 stimulus with the same amplitude and duration as the S1 was applied to the junction of the EPI to MIDDLE cells. This action led to the initiation of a re-entrant excitation wave that then rotated through the tissue. However, the S2 had variable spatial sizes (width 0.45 mm, length varies between 0 and 75 mm). A S2 stimulus with sufficient spatial size (S2 size) in 2D tissue is required to provide an adequate re-entrant pathway, which is dependent on the wavelength (wavelength = ERP × CV) of re-entry. In order to evaluate the critical size of the re-entrant pathway, we estimated the minimum length of S2 stimulus. The minimum length was measured as the smallest length which supported the formation of re-entry by decreasing it from 75 to 0 mm with decrement of 0.15 mm. This minimum length gives an indication of the susceptibility of the tissue to sustain re-entry, i.e., the larger the minimum length, the more difficult for the initiation of re-entry [37]. For such an initiated re-entrant excitation waves, effects of amiodarone on their dynamical behaviors were evaluated in the SQT3 condition.

## Results

### Effects of amiodarone on SQT3 in the cell model

**Fig. 2:**
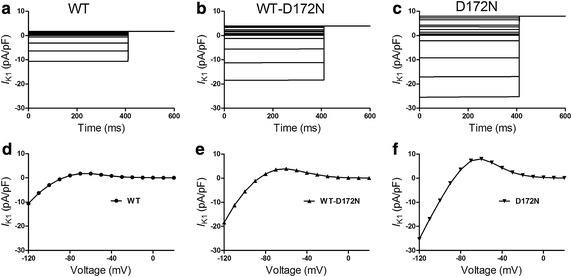
Simulated current–voltage (*I*–*V*) relationships. **a**–**c** Currents traces for WT, WT-D172N and D172N *I*_K1_ elicited by 400 ms depolarizing voltage steps from −120 to +20 mV and from a holding potential of −60 mV. **d**–**f** Current–voltage relationships. The current amplitude of the WT-D172N and D172N mutant conditions is significantly larger than that of the WT condition

Figure 2 shows the results of simulated current–voltage (*I*–*V*) for WT and mutant *I*_K1_ channels. In simulations, *I*_K1_ was elicited by 400 ms depolarizing voltage steps from −120 to +20 mV and from a holding potential of −60 mV, with current traces for WT, WT-D172N and D172N *I*_K1_ current shown respectively in Fig. 2a–c. Figure 2d–f shows current–voltage (*I*–*V*) data for WT, WT-D172N and D172N conditions. The reversal potential of the *I*–*V* relations for WT and mutant (WT-D172N and D172N) *I*_K1_ were not significantly different. However, outward *I*_K1_ current differed markedly between WT and mutant channels: it was significantly greater for WT-D172N and D172N conditions than for WT condition. These features are consistent with those experimental data reported previously [2].

**Fig. 3:**
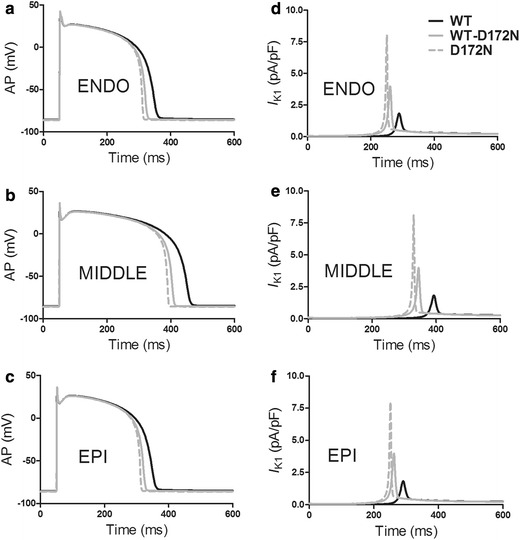
Simulations of APs together with the corresponding time course and amplitude of *I*_K1_ in the WT, WT-D172N and D172N conditions. **a**, **d** APs and *I*_K1_ for ENDO cells. **b**, **e** APs and *I*_K1_ for MIDDLE cells. **c**, **f** APs and *I*_K1_ for EPI cells

Changes in *I*_K1_ due to the *KCNJ2* D172N mutation abbreviated human ventricular APD as shown in Fig. 3. Figure 3a–c shows AP waveforms *V* (mV) versus time *t* (ms). The measured APD_90_ was 302, 406 and 304 ms for the ENDO, MIDDLE and EPI cell, respectively, in the WT condition, which were shortened, respectively, to 273, 357 and 274 ms for the WT-D172N condition and to 261, 341 and 262 ms for the D172N condition. The abbreviated APD resulted from an increased *I*_K1_ during the AP repolarization phase as shown in Fig. 3d–f.

**Fig. 4:**
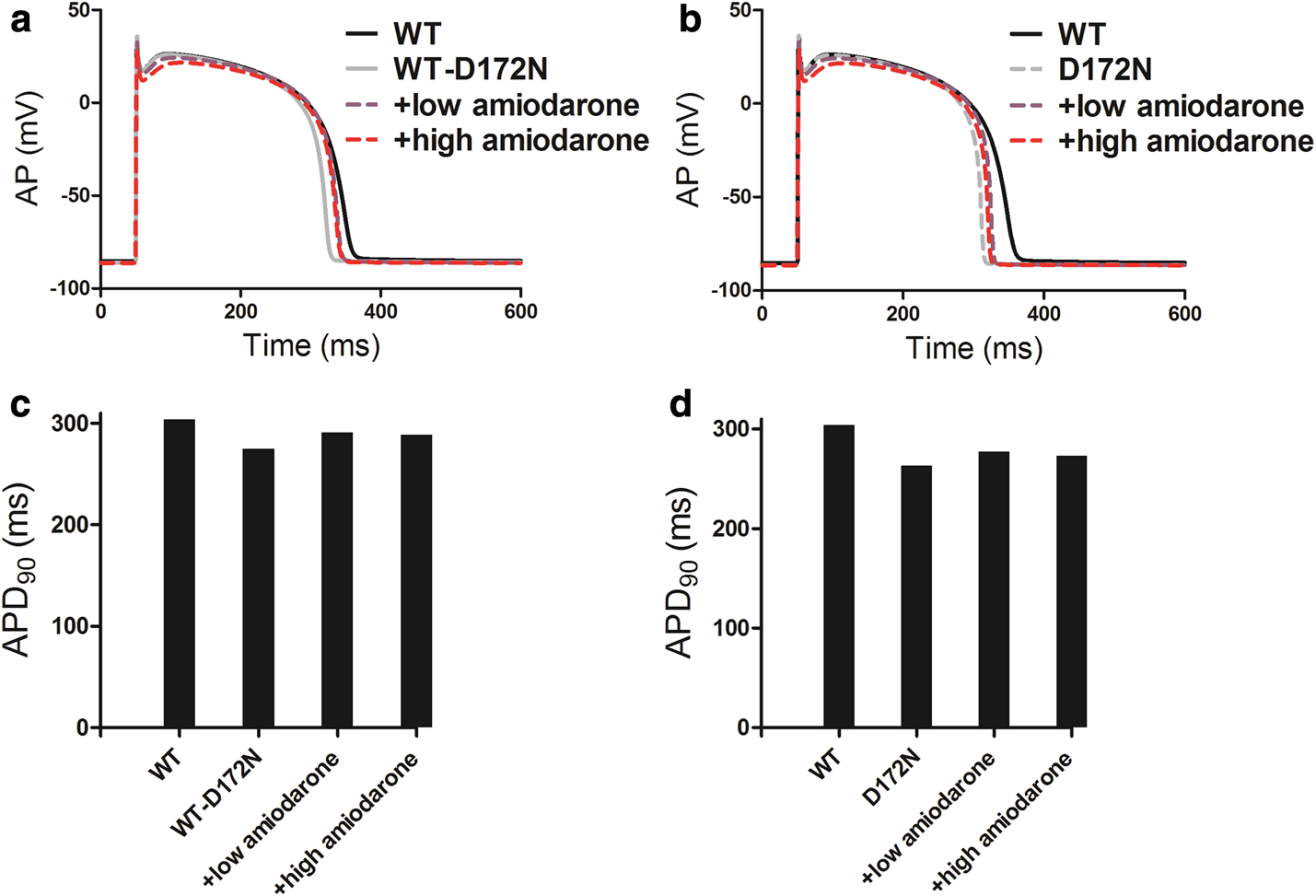
Effects of low and high doses of amiodarone on human ventricular EPI cell. **a**, **c** APs under the WT-D172N condition in the presence of amiodarone, and corresponding APD_90_ histogram. **b**, **d** APs under the D172N condition in the presence of amiodarone, and corresponding APD_90_ histogram

The effects of different doses of amiodarone on the AP characteristics for the human ventricular EPI cells are shown in Fig. 4a (WT-D172N condition) and b (D172N condition), and the corresponding APD_90_ are plotted in Fig. 4c and d. The measured APD_90_ was prolonged from 275 ms in WT-D172N to 291 and 289 in the presence of low and high doses of amiodarone, respectively, and from 263 ms in D172N condition to 277 and 273 ms in the presence of low and high doses of amiodarone. Additional simulations on ENDO and MIDDLE cells (data not shown) showed similar effects of amiodarone to those seen with the EPI ventricular AP waveform.

**Fig. 5:**
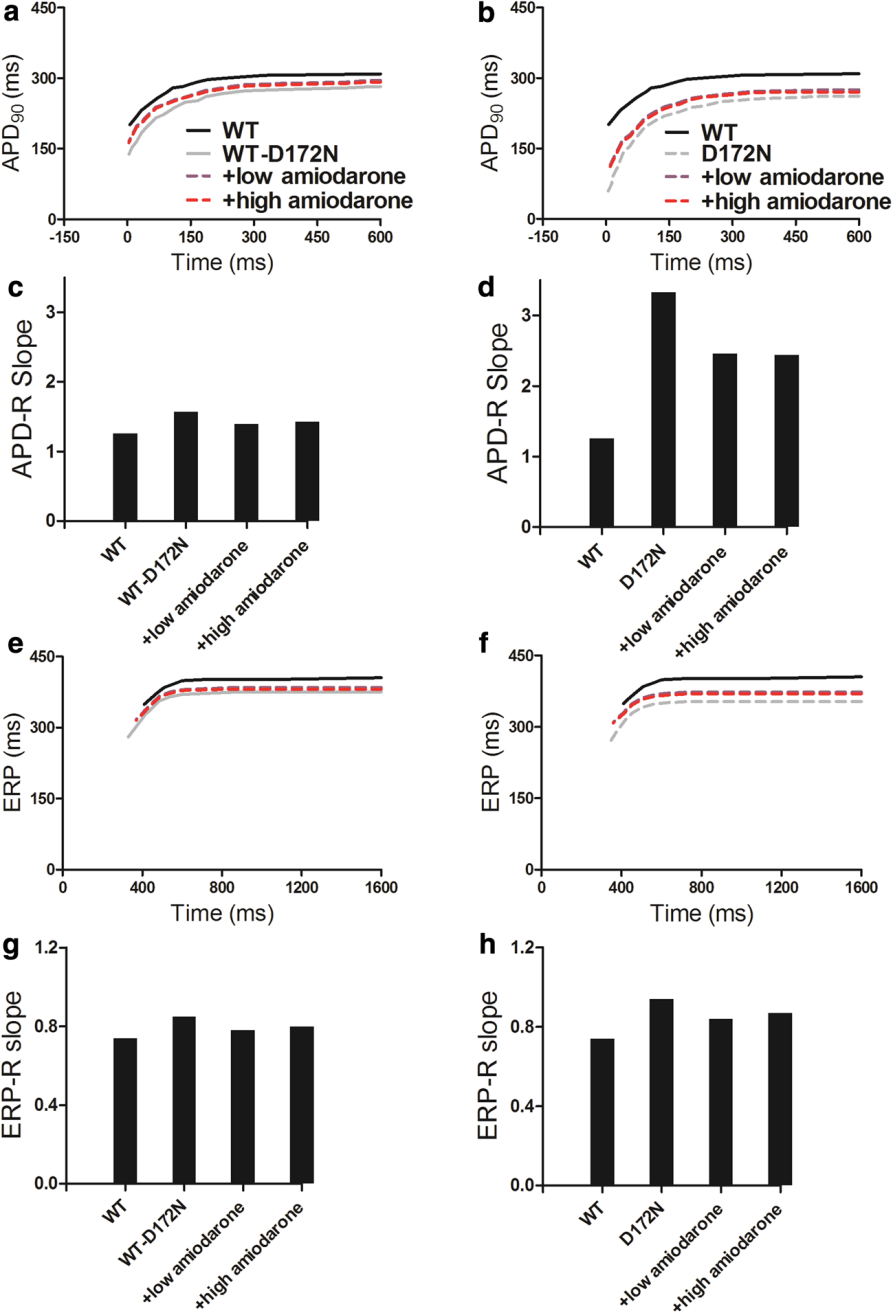
Rate-dependent APD restitution curves and ERP restitution curves for EPI cells under the WT, WT-D172N and D172N conditions, and in the presence of amiodarone. **a**, **b** APD restitution curves under the WT-D172N and D172N conditions, respectively. **c**, **d** Measured slopes of APD restitution curves under the WT-D172N and D172N conditions, respectively. **e**, **f** ERP restitution curves under the WT-D172N and D172N conditions, respectively. **g**, **h** Measured slopes of ERP restitution curves under the WT-D172N and D172N conditions, respectively

Rate-dependent effects of amiodarone on the APD of EPI cells was shown in Fig. 5a and b for the computed APD restitution (APD-R) curves for WT and mutation conditions. Across the range of diastolic intervals (DI) studied, the measured APD_90_ was increased in the presence of amiodarone. In addition, the APD-R relationships were attenuated by the action of amiodarone, as indicated by the decreased maximal slope of the APD-R curves (Fig. 5c, d). The measured ERP was also increased in the presence of amiodarone. The ERP reduction was also rate-dependent in Fig. 5e and f. Across a range of BCLs, the measured ERP in the presence of amiodarone was increased. Actions of amiodarone also attenuated the ERP-R curves, as indicated by the decreased maximal slope of the ERP-R curve (Fig. 5g, h) in the presence of amiodarone. As a decreased steepness of APD-R and ERP-R curves are believed to be associated with stability of re-entry [43], indicating anti-arrhythmic effects of amiodarone on SQT3.

### Effects of amiodarone on SQT3 in the 1D fiber model

**Fig. 6:**
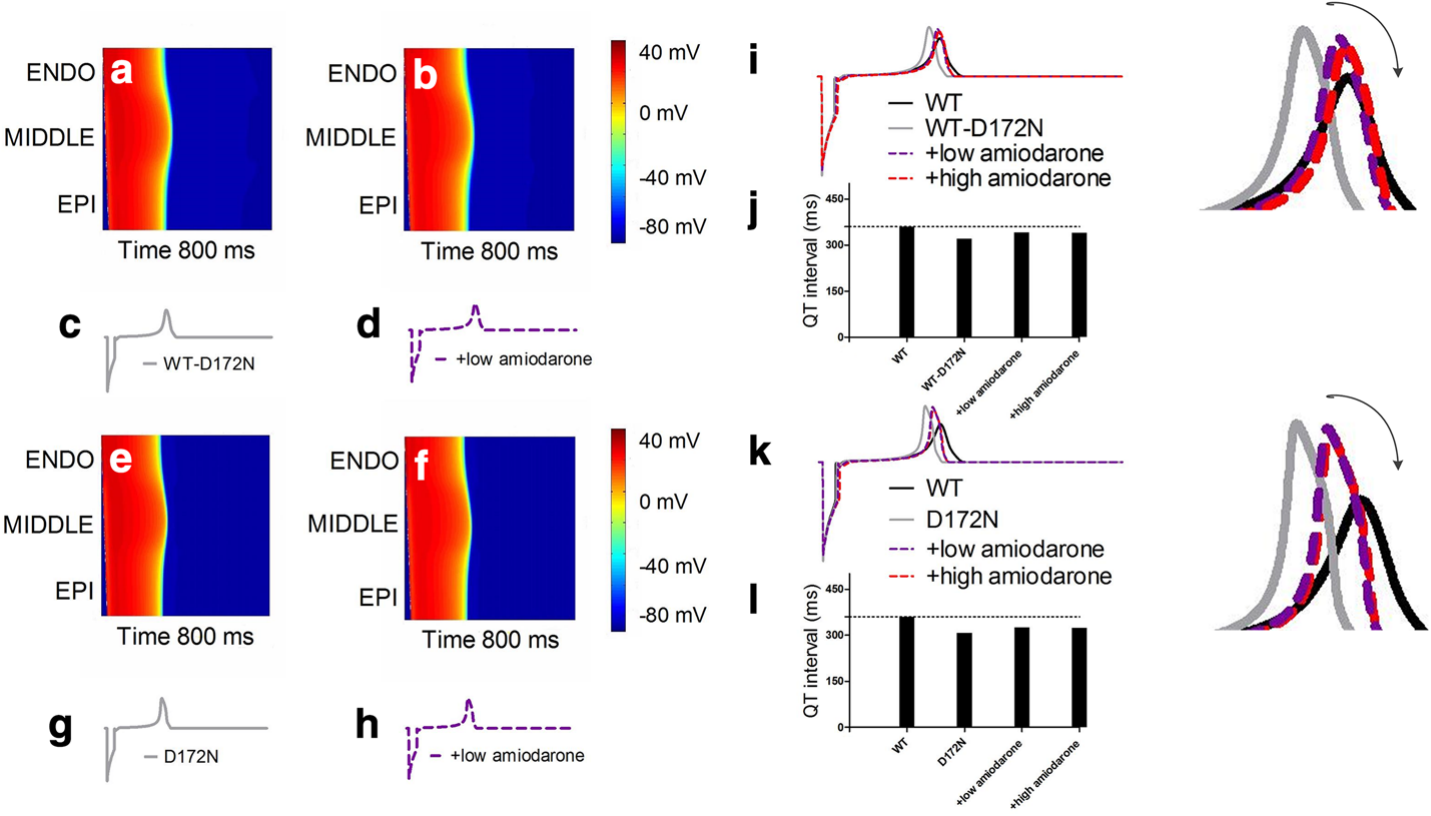
Space–time plot of AP propagation along a 1D fiber and the pseudo-ECGs. **a**, **b** *Color* mapping of membrane potential of cells in the WT-D172N and a low dose of amiodarone conditions, respectively. Space runs vertically from the ENDO end at the *top* to the EPI end at the *bottom*. Time runs* horizontally* (*left to right*). **c**, **d** The corresponding pseudo-ECGs derived from the propagating excitation wave for the WT-D172N and a low dose of amiodarone conditions, respectively. **e**, **f** *Color* mapping of membrane potential of cells in the D172N and a low dose of amiodarone conditions, respectively. **g**, **h** The corresponding pseudo-ECGs derived from the propagating excitation wave for the D172N and a low dose of amiodarone conditions, respectively. **i**, **k** Superimposed pseudo-ECGs for the WT-D172N and D172N conditions, respectively. **j**, **l** Associated QT intervals

Using a 1D-fiber model, a pseudo-ECG was computed for the WT, WT-D172N, D172N and actions of amiodarone conditions (Fig. 6). The results are shown in Fig. 6a, b, e and f for an excitation wave propagating from ENDO towards MIDDLE and EPI parts of the fiber. Amiodarone delayed the repolarization phase both in the WT-D172N and D172N conditions. These changes of the excitation wave were also reflected in the computed pseudo-ECGs as shown in Fig. 6c, d, g and h for the WT-D172N, D172N and amiodarone-in-action conditions respectively. To quantify the effects, we calculated the QT intervals extracted from the simulation data (Fig. 6i–l). In simulations, the mutation shortened APD, leading to abbreviated QT interval (322 ms for WT-D172N condition), which is similar to clinical observation (315 ms for the proband and 320 ms for her father) [2]. The simulated mutation produced a taller T-wave amplitude, which is also similar to clinical observation [2]. The pseudo-ECGs show an increment in the QT interval in the presence of low and high doses of amiodarone, which changed from 322 ms in the WT-D172N condition to 341 and 340 ms, respectively. For the same condition, the QT interval was prolonged from 308 ms in the D172N condition to 325 and 324 ms, respectively. These QT intervals were prolonged, but were not within the normal physiological range (from 360 to 440 ms). The simulated small QT interval prolongation was consistent with the clinical study [18] in which the patient remained short QT after use of amiodarone. T-wave amplitude in the presence of amiodarone under WT-D172N and D172N conditions was decreased as shown in Fig. 6i and k.

**Fig. 7:**
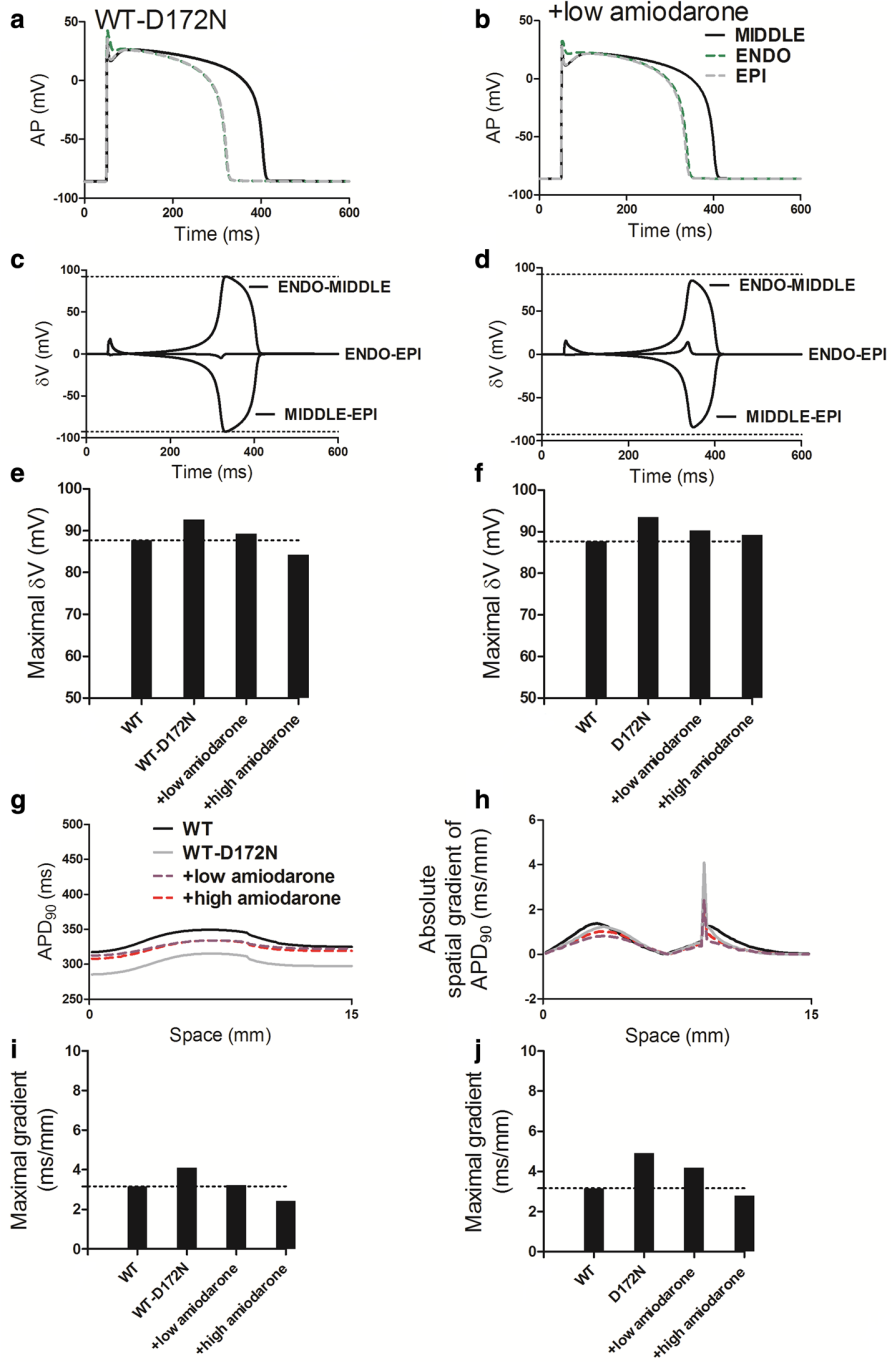
Membrane potential heterogeneity (*δV*) between single ENDO, MIDDLE and EPI cells and transmural APD_90_ distribution and its spatial gradient in 1D fiber model for the WT, WT-D172N, D172N and amiodarone-in-action conditions. **a**, **b** ENDO, MIDDLE and EPI APs in WT-D172N and WT-D172N + low amiodarone conditions. **c**, **d** Corresponding plots of *δV* against time for the two conditions.* Horizontal dashed lines* mark peak ENDO-MIDDLE and MIDDLE-EPI *δV* in WT-D172N condition. **e**, **f** Maximal *δV* observed during repolarization (ENDO-MIDDLE) under WT-D172N and D172N conditions in the presence of amiodarone, respectively.* Horizontal dashed lines* mark peak ENDO-MIDDLE *δV* in WT condition. **g** Spatial distribution of APD_90_ on 1D fiber model in WT, WT-D172N and amiodarone-in-action conditions. **h** Spatial gradient of APD_90_ on 1D fiber model in WT, WT-D172N and amiodarone-in-action conditions. **i**, **j** The maximal spatial gradient of APD_90_ under WT-D172N and D172N conditions in the presence of amiodarone, respectively

An increase in T-wave amplitude during simulated SQTS has been attributed to an increased spatial gradient in membrane potential [36, 37] and an inverse effect might explain the decrease T-wave amplitude seen in Fig. 6 of this study. Therefore, we examined effects of amiodarone on the membrane potential heterogeneity (*δV*) which was calculated as the difference between APs among three cell types. Via cell-to-cell electrical interaction, *δV* (in the temporal domain) is smoothed in space producing a spatial gradient ∇*V* (in the spatial domain), which contributes to the computed pseudo-ECG as described in Eq. 4. Figure 7a and b show simulated ENDO, MIDDLE and EPI APs with WT-D172N and WT-D172N + low amiodarone conditions whilst Fig. 7c and d show corresponding time-course plots of the *δV* during APs between ENDO, MIDDLE and EPI cells. The maximal *δV* between both ENDO-MIDDLE and MIDDLE-EPI APs was smaller with the use of amiodarone than before (Fig. 7e, f). The actions of amiodarone also augmented APD_90_ dispersion in the fiber model with the electrotonic interaction between cells. Figure 7g shows the spatial distribution of APD_90_ for WT, WT-D172N and amiodarone-in-action conditions. The absolute spatial gradient of APD_90_ was found to be augmented, as shown in Fig. 7h. The maximal gradient across the 1D fiber was smaller with the use of amiodarone than before (Fig. 7i, j). The D172N mutation augmented the APD dispersion at the junction region between the MIDDLE and EPI regions, whilst amiodarone attenuated the APD dispersion across the 1D strand, which also led to the decreased T-wave amplitude.

**Fig. 8:**
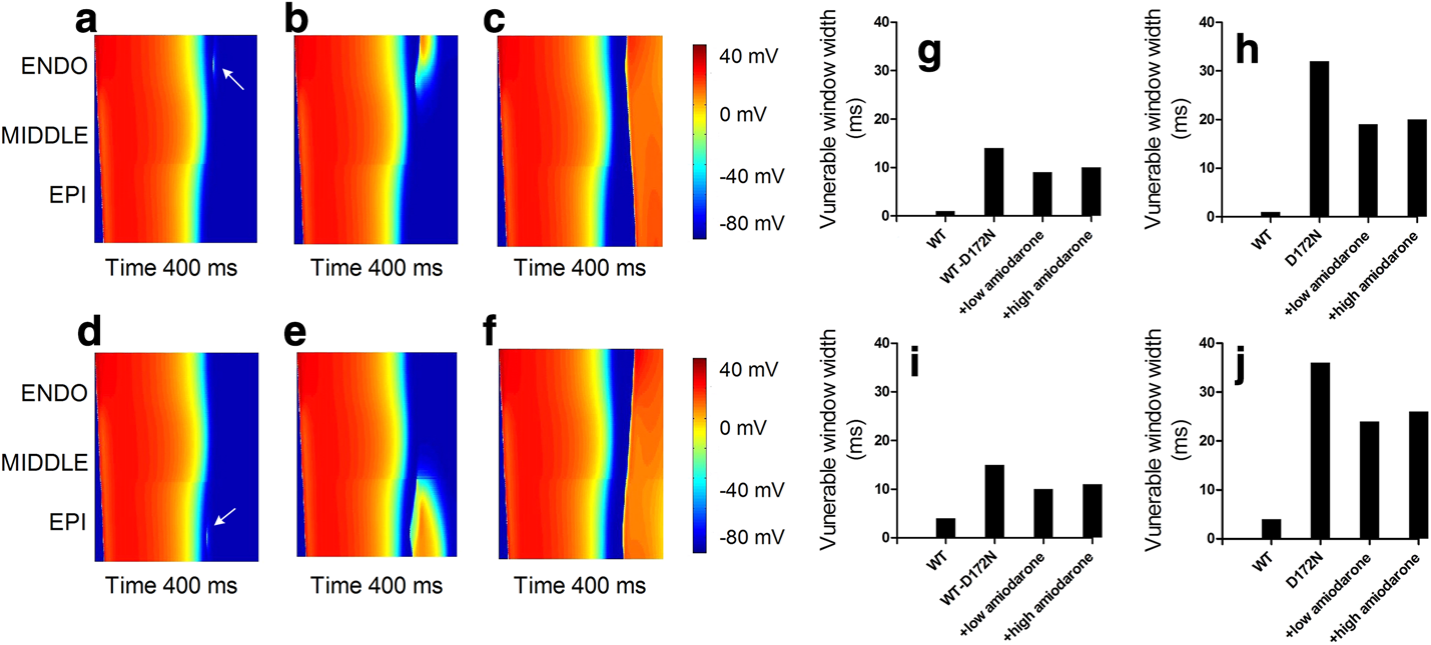
Space–time plots of propagating conditioning excitation wave and response of the tissue to a premature stimulus in the WT condition. Membrane potentials of cells are mapped onto a color spectrum. Space runs vertically from the ENDO end at the *top* to the EPI end at the *bottom*. Time runs* horizontally* (*left to right*). **a–c**, **g**, **h** Premature stimulus applied at ENDO part at: **a** 335 ms, bidirectional block. **b** 341.5 ms, unidirectional block. **c** 350 ms, bidirectional conduction. **g** Vulnerability of the tissue in WT-D172N and amiodarone-in-action conditions. **h** Vulnerability of the tissue in D172N and amiodarone-in-action conditions. **d**–**f**, **i**, **j** Premature stimulus applied at EPI part at: **d** 332 ms, bidirectional block. **e** 340 ms, unidirectional block. **f** 350 ms, bidirectional conduction. **i** Vulnerability of the tissue in WT-D172N and amiodarone-in-action conditions. **j** Vulnerability of the tissue in D172N and amiodarone-in-action conditions

Simulations were also performed to determine whether or not amiodarone decreased susceptibility of tissue to ventricular arrhythmogenesis. Using a 1D fiber model we quantified tissue’s temporal vulnerability to unidirectional conduction in response to a premature stimulus for the amiodarone-in-action condition. Figure 8a–c shows the conditioning excitation wave and the response of the tissue to a premature stimulus applied at ENDO part of the fiber (marked by the arrow). In Fig. 8a, the premature stimulus was applied early (i.e., at time = 335 ms after the previous excitation wave) so that the tissue surrounding the stimulus site did not have enough time to recover for re-excitation to occur. As a consequence, a bidirectional block was observed. In Fig. 8b, the premature stimulus was applied within the vulnerable window (at time = 341.5 ms) that produced a unidirectional conduction block. In Fig. 8c, the premature stimulus was applied after the vulnerable window (at time = 350 ms) and, consequently, bidirectional conduction was observed. Figure 8g and h show the width of the vulnerable window during which a premature stimulus applied at the ENDO part of the fiber resulted in the unidirectional block under WT, WT-D172N, D172N and amiodarone-in-action conditions. Simulations were also performed in which the premature stimulus was applied to the EPI rather than the ENDO part of the fiber (Fig. 8d–f). Figure 8d–f represent bidirectional conduction block (at time = 332 ms), unidirectional block (at time = 340 ms) and bidirectional conduction (at time = 350 ms). Effects of amiodarone on temporal vulnerability in WT-D172N and D172N mutations are shown in Fig. 8i and j. These simulation results show clearly that the tissue’s temporal vulnerability is decreased by amiodarone.

### Effects of amiodarone on SQT3 in the 2D tissue model

**Fig. 9:**
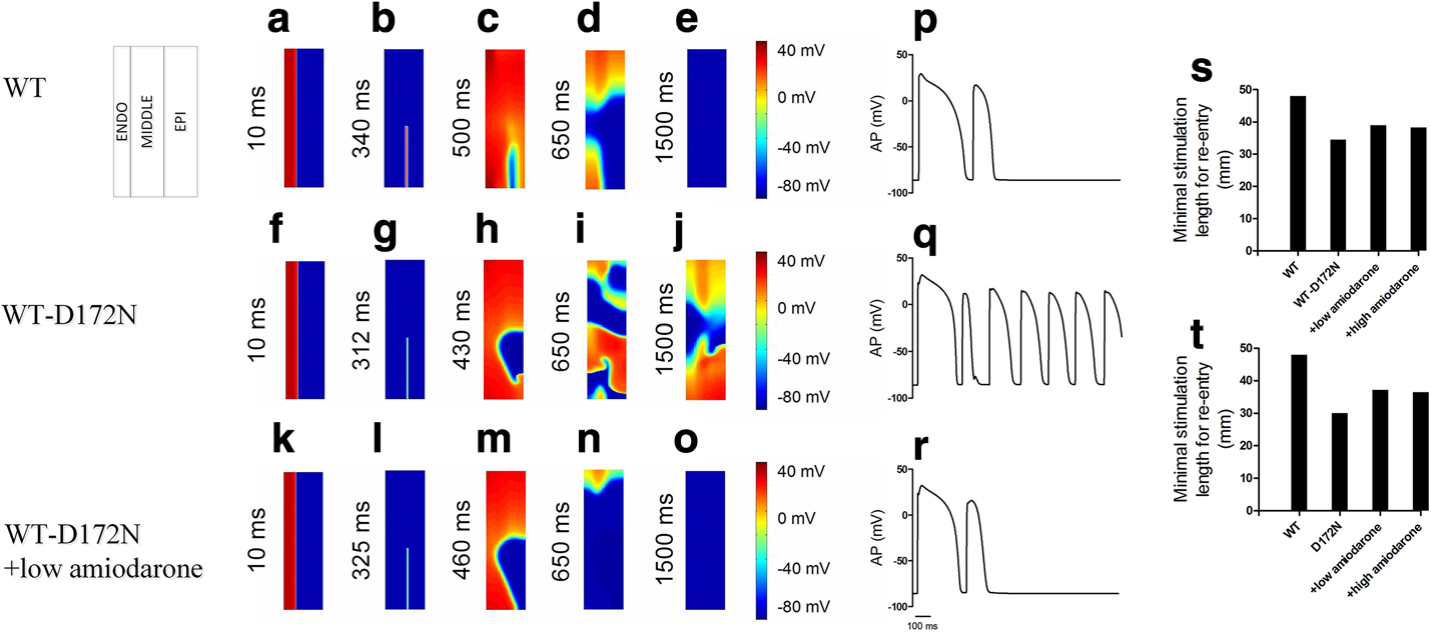
Snapshots of initiation and conduction of re-entrant excitation wave in a 2D model of transmural ventricle tissue under the WT, WT-D172N and amiodarone-in-action conditions. **a**, **f**, **k** A planar wave generated by S1 stimulus at the ENDO end, which propagates towards the MIDDLE and EPI regions. Snapshots at time = 10 ms. **b**, **g**, **l** S2 was applied to the MIDDLE-EPI junction region during the vulnerable window of the tissue. **c**, **h**, **m** Developed re-entrant excitation waves. **d**, **i**, **n** Developed re-entrant excitation waves. Snapshots at time 650 ms. **e**, **j**, **o** Snapshots of the re-entrant excitation waves. Re-entry was prevented in the presence of amiodarone. Snapshots at time 1500 ms. **p**, **q**, **r** Evolution of the AP of an EPI cell under the WT, WT-D172N and WT-D172N + low amiodarone conditions, respectively. **s**, **t** The measured minimum spatial length of S2 that provides a sufficient substrate for the formation of a re-entrant circuit under WT-D172N and D172N conditions in the presence of amiodarone, respectively

Using an idealized 2D sheet model, we proceeded to measure the minimum spatial size of a premature S2 stimulus that provides a sufficient substrate size for sustaining re-entry initiated for SQT3 and amiodarone-in-action conditions. Results are shown in Fig. 9, in which patterns of re-entrant excitation waves are represented via pseudo-color mapping of transmembrane voltage. During the simulation, the S1 stimulus was applied to the ENDO end to evoke a planer excitation wave that propagated towards the MIDDLE and EPI regions (Fig. 9a, f, k). After a time delay (WT: 340 ms; WT-D172N: 312 ms; WT-D172N + low amiodarone: 325 ms), a S2 stimulus was applied to the MIDDLE-EPI junction region that produced a unidirectional conduction, forming a re-entrant excitation wave (Fig. 9b, g, l). The induced re-entrant excitation self-terminated for the WT condition (Fig. 9c–e), but sustained for the WT-D172N mutation condition (Fig. 9h–j). It is attributable to the shortening of APD due to the mutation, decreasing the wavelength of the ventricular excitation wave. With the use of amiodarone, re-entrant excitation wave self-terminated as shown in Fig. 9m–o. Figure 9p–r show a recording of the evolution of the AP of an EPI cell in the 2D sheet model under the WT, WT-D172N and low dose of amiodarone conditions, respectively. The measured minimum spatial length of S2 stimulus required for re-entry under WT-D172N and D172N conditions in the presence of amiodarone is shown in Fig. 9s and t, respectively. Prolongation of APD due to the actions of amiodarone increased the wavelength of the ventricular excitation wave, and thus increased the minimal length of S2 stimulus. This result supports the notion that in SQT3 tissue with the use of amiodarone, re-entrant excitation waves is less likely to occur.

## Discussion

### Summary of the major findings

The proband in whom the *KCNJ2* D172N mutation was identified was heterozygotic for the SQT3 mutation (WT-D172N) [2]. She had a QTc interval of 315 ms, whereas her father had a QTc interval of 320 ms [2]. It is of particular note that with the use of amiodarone in the WT-D172N condition, mimicking the effects on the heterozygous state of the proband, we found that the simulated QT interval extended from 322 to 341 ms in the low-dose amiodarone condition and 340 ms in the high-dose condition. The QT interval extended from 308 ms in the D172N condition to 325 ms in the low-dose amiodarone condition and 324 ms in the high-dose condition. In addition, the risk of arrhythmogenesis as measured by the vulnerability of tissue was decreased in the presence of both low and high doses of amiodarone. Our simulation results indicate that the amiodarone (i) extended the APD_90_ in the SQT3 condition and decreased susceptibility to arrhythmia; (ii) extended the QT interval on the pseudo-ECG and reduced the T-wave amplitude; (iii) decreased the maximal voltage heterogeneity (*δV*) during APs, which contributed to the decreased T-wave amplitude; (iv) prolonged cell APD_90_ across the 1D strand and decreased maximal dispersion of APD_90_, which also subsequently led to the decreased T-wave amplitude; (v) prevented re-entrant excitation waves and increased the minimum substrate size of tissue required to maintain re-entrant excitation waves. These changes due to the presence of amiodarone indicated its anti-arrhythmic effects in SQT3 condition.

### Relevance of the study

SQT3 produces changes on the QT interval and T-wave, caused by a shortening of total repolarization time, which are in agreement with the previous studies [2, 10]. Those studies investigated the *I*_K1_ current underlying the changes in the pseudo-ECG caused by a D172N mutation in the SQT3 condition. However, the present study first assessed the pharmacological effect of amiodarone on ventricular excitation associated with SQT3. Our simulations were able to reproduce the known effects of D172N mutation on the electrophysiological activity of the ventricles as shown in the literature [2], which provide the first step towards the validation of the use of the cardiac models in assessing the effects of amiodarone on SQT3.

### Significance of the study

Our simulations showed the anti-arrhythmic effects of amiodarone on SQT3. This was done by thoroughly analyzing the functional effects of amiodarone at the cell and tissue levels. First, the action of amiodarone was simulated by using a single pore block theory, as in the previous studies [44, 45]. Then, the effects of amiodarone were investigated in a 1D ventricular model, using which pseudo-ECGs were simulated. Finally, effects of amiodarone on re-entrant excitation wave dynamics in SQT3 condition were quantitatively assessed. Our results showed that both of low and high doses of amiodarone exhibited anti-arrhythmic effects for SQT3.

The results demonstrated that the presence of amiodarone led to QT interval prolongation and a reduction in the T-wave amplitude. As expected, the QT interval prolongation is explained by an APD prolongation, whereas the decrease in the T-wave amplitude correlates with reduced dispersion in repolarization as demonstrated by decreased dispersion of the APD_90_ across the 1D fiber model.

In the SQT3 setting, ventricular fibrillation could be elicited by programmed electrical stimulation. It was hypothesized that APD and ERP abbreviation are factors contributing to an increased risk of re-entrant arrhythmias. Amiodarone prevented re-entrant excitation waves. This is attributable to the APD prolongation, which increased the wavelength of excitation waves, and therefore, increased the critical mass of tissue necessary to accommodate re-entrant excitation waves, suppressing the maintenance of re-entrant excitation waves. Thus, re-entrant excitation wave in the presence of amiodarone becomes more difficult to be initiated and sustained. This simulation result was consistent with no further syncope or palpitations in clinical study [18].

In this study, due to the scarcity of accurate experimental models of SQT3 and of in vitro pharmacological data of SQT3, we are currently unable to compare our simulation results with experiments for validation. However, we used the well-established ten Tusscher et al. [21] model for simulating human ventricular APs along with the recent SQT3 *I*_K1_ model developed by our group [10]. These models were validated previously. With regard to amiodarone effects, although there are no direct experimental data for us to compare, results from the clinical study [18] have demonstrated the beneficial effects of amiodarone on SQTS patients. Our simulation data are in accordance with the clinical phenomenon [18], indicating the anti-arrhythmic effect of amiodarone in SQT3 condition.

### Limitations of the study

Limitations of the ten Tusscher et al. [21] model have been discussed in detail elsewhere. Although such a model is not as complete as the newest models [33], it is sufficient for the purpose of this study as the simulated effect of amiodarone on SQT3 is in accordance with the clinical phenomenon [18]. The biophysical approach, which we adopt here, has also been used by several other groups (see, e.g. [38, 46, 47]).

Although we considered intercellular electrical coupling with each distinct cell type, the 1D and 2D idealized models cannot represent a realistic anatomical structure, which might influence the maintenance of ventricular arrhythmias. The structure of realistic anatomical tissue is much more complex than the idealized isotropic tissue studied here [48]. The idealized model, which was used to simulate re-entrant excitation waves, lacks inclusion of Purkinje fibers, which may play a significant role in arrhythmogenesis in SQTS. Furthermore, the model did not consider the effect of the atria, mechano-electric coupling and feedback, tissue deformation and coronary flow, which feasibly might influence re-entrant excitation waves.

Regarding the experimental conditions, many factors that may affect the binding of a drug to ion channels are increasingly being modeled, including oxygen, the concentration of ions, temperature and pH [49]. However, pharmaceutical screening does not usually record sufficient data to evaluate the dynamic changes caused by drugs [45]. For conditions with variable pacing rates, conductance block approximation may be insufficient for simulation of realistic amiodarone actions.

Another limitation is the use of data from animal models instead of human to mathematically describe the effects of amiodarone on the ionic currents as listed in Table 1. Limitations of data from animal models of disease in nonclinical safety testing include a lack of historical control, heterogeneity in disease expression, a limited life span and confounding effects of the disease. However, little information is known about the effects of amiodarone on the specific ionic currents in human. Therefore, for future work, integration of experimental data for the different ion channels of human heart acquired at body temperature when become available could provide a better insight into the effects of amiodarone on SQT3.

## Conclusions

In this study, we presented a simulation study showing the effects of amiodarone on SQT3. It was shown that amiodarone altered the profiles of APs and pseudo-ECGs, as well as the dynamics of re-entrant excitation waves. Amiodarone produced QT interval prolongation (owing to APD prolongation), decreased APD dispersion and membrane potential dispersion (*δV*), which contributed to the decreased T-wave amplitude. Additionally, amiodarone increased ERP, reduced vulnerable window and prevented re-entrant excitation waves. Our results demonstrate anti-arrhythmic effects of amiodarone on SQT3 and suggest that amiodarone may be a potential pharmacological agent for treating SQT3 patients.

## Additional file

**Additional file 1.** An appendix showing *I*_K1_ model equation parameters for WT, WT-D172N and K1 D172N conditions.

## Abbreviations

SQTS: short QT syndrome
SCD: sudden cardiac death
*I*_K1_: inward rectifier potassium channel current
APD: action potential duration
ERP: effective refractory period
APs: action potentials
1D: one-dimensional
2D: two-dimensional
*δV*: maximal voltage heterogeneity
SQT3: *KCNJ2*-lined short QT syndrome
QTc: corrected QT interval
ECG: electrocardiogram
APD-R: APD restitution
ERP-R: ERP restitution
ICD: implantable cardioverter defibrillator
ENDO: endocardial
MIDDLE: mid-myocardial EPI: epicardial
WT: wild-type
IC_50_: half maximal inhibitory concentration
nH: Hill coefficient
*I*_NaL_: late sodium current
BCL: basic cycle length
HH: Hodgkin–Huxley
CV: conduction velocity
*I*–*V*: current–voltage
DI: diastolic interval
