# Supplementary material for: Effects of amiodarone on short QT syndrome variant 3 in human ventricles: a simulation study
Source: Biomed Eng Online. 2017 Jun 7;16:69. doi: 10.1186/s12938-017-0369-0 (PMC5463381; doi:10.1186/s12938-017-0369-0)
Supplement: Supplementary file 1 — Additional file 1. An appendix showing I K1 model equation parameters for WT, WT-D172N and K1 D172N conditions. [file 12938_2017_369_MOESM1_ESM.docx]

# Effects of Amiodarone on Short QT Syndrome Variant 3 in Human Ventricles: A Simulation Study

Cunjin Luo^1^, Kuanquan Wang^1^* and Henggui Zhang^1,2,3^*

*Correspondence

[wangkq@hit.edu.cn](mailto:wangkq@hit.edu.cn) (Kuanquan Wang)

[H.Zhang-3@manchester.ac.uk](mailto:H.Zhang-3@manchester.ac.uk) (Henggui Zhang)

^1^School of Computer Science and Technology, Harbin Institute of Technology (HIT), Harbin 150001, China

*I*_K1_ formulations [[1](#_ENREF_1)] were modified based on the extant experimentally determined properties of Kir2.1 D172N channels, which are described below:

WT:

$$\alpha_{K1}=\frac{0.07}{1+e^{0.017(V-E_{K}-200.2)}}$$

$$\beta_{K1}=\frac{3e^{0.0003(V-E_{K}+100.2)}+e^{0.08(V-E_{K}-8.7)}}{1+e^{-0.024(V-E_{K})}}$$

$$G_{K1}=4.8\frac{nS}{pF}$$

WT-D172N:

$$\alpha_{K1}=\frac{0.1}{1+e^{0.023(V-E_{K}-199.9)}}$$

$$\beta_{K1}=\frac{3e^{0.0002(V-E_{K}+100.4)}+e^{0.07(V-E_{K}-9.8)}}{1+e^{-0.021(V-E_{K})}}$$

$$G_{K1}=6.27\frac{nS}{pF}$$

D172N:

$$\alpha_{K1}=\frac{0.1}{1+e^{0.05(V-E_{K}-199.9)}}$$

$$\beta_{K1}=\frac{3e^{0.0002(V-E_{K}+100.1)}+e^{0.08(V-E_{K}-10.3)}}{1+e^{-0.006(V-E_{K})}}$$

$$G_{K1}=11.32\frac{nS}{pF}$$

[1] I. Adeniran, A. El Harchi, J.C. Hancox, H. Zhang, Proarrhythmia in KCNJ2-linked short QT syndrome: insights from modelling, Cardiovasc Res 94(1) (2012) 66-76.
